# The Endocrine Role of the Skeleton

**DOI:** 10.1155/2015/265151

**Published:** 2015-04-02

**Authors:** Martina Rauner, Amélie Coudert, Cristina Sobacchi, Andrea Del Fattore

**Affiliations:** ^1^Medical Faculty of the Technische Universität Dresden, 01309 Dresden, Germany; ^2^Institut National de la Santé et de la Recherche Médicale U1138, Centre de Recherche des Cordeliers, 75006 Paris, France; ^3^UOS/IRGB, Milan Unit, National Research Council (CNR), 20138 Milan, Italy; ^4^Humanitas Clinical and Research Center, 20089 Rozzano, Italy; ^5^Bambino Gesù Children's Hospital, 00146 Rome, Italy

In recent years, many advances have been made in the understanding of the role of the skeleton in whole body pathophysiology.

The original vision of the bone as a static tissue with limited functions is now completely abolished. Indeed it is now well established that bone is a dynamic connective tissue subjected to a continuous process of resorption and formation. This activity is crucial, since correct bone homeostasis, skeleton integrity, and mechanical properties rely on a perfect coupling between osteoclasts and osteoblasts functions. Indeed recent studies demonstrated that the bone remodelling process is an important regulator of many functions not only limited to the bone, but also correlated with the whole body physiology.

In this special issue, experts in the bone field addressed the interplay between bone and energy metabolism, immune system, male fertility, and kidney and documented how bone has emerged as an endocrine “gland.”

In particular, osteocalcin is indicated as a key factor mediating the endocrine functions of bone, with special focus on glucose metabolism and male fertility. J. Shao et al. described how osteocalcin targets *β*-cells in the pancreas and adipocytes to regulate insulin production and sensitivity and Leydig cells to control testicular function. Moreover the authors reviewed the mechanisms regulating osteocalcin production and activation, with particular attention to 1*α*,25-dihydroxyvitamin D3 and vitamin K, and reported studies in humans supporting the relevance of osteocalcin in energy metabolism and highlighting differences between men and mice as well as aspects requiring further investigation. M. F. Faienza et al. analyzed the bone-pancreas loop, focusing the attention not only on osteocalcin, but also on other proteins, such as osteoprotegerin, vitamin D, gastric inhibitory polypeptide (GIP), and adiponectin, involved in the regulation of insulin function and glucose metabolism. Antiresorptive drugs might impact the bone-pancreas interplay; however conflicting results exist on this aspect and deserve further efforts from the scientific community. In particular, the development of new drugs simultaneously targeting the skeleton, glucose metabolism, and the adipose tissue can be envisaged. Besides the effects of bone osteocalcin on male fertility, the endocrine role of oestrogens on human male skeleton was also discussed in this special issue in the paper by V. Rochira et al. The authors described the effects exerted by oestrogens in the physiological events occurring in male bone throughout life such as longitudinal skeletal growth, skeletal proportion, achievement of peak bone mass, and maintenance of bone mineral density. Moreover the authors discussed clinical aspects of oestrogen deficiency.

In separate chapters, the interplay between bone and kidney is presented. Y. Takei et al. described how bone homeostasis is finely regulated by a complex mechanism including FGFs (*fibroblast growth factors*)/FGF receptors signalling. In particular they emphasized how FGF23, expressed by osteocytes/osteoblasts, reduces the levels of serum phosphate and 1,25(OH)_2_D_3_ and the relevance of its coreceptor *α*Klotho, produced by kidney distal tubular cells, in the physiologic regulation of mineral metabolism. In a clinical study S. Rotondi et al. investigated whether a soluble form of Klotho could represent a marker of renal damage of CKD- (chronic kidney disease-) MBD (mineral bone disorder), indicative of the cross-talk between bone and kidney, and concluded that soluble Klotho could be considered an early marker of mineral metabolism impairment in renal disease.

Finally, bone diseases, such as osteopetrosis and osteoporosis, are revisited with the special focus on their relevance for the endocrine role of the skeleton and on the relationship with other diseases, respectively. Indeed, A. E. Coudert et al. described how osteoclast dysfunctions lead to osteopetrosis and have allowed shedding some lights on several aspects of the bone biology that were not well known, discovering the interaction between bone and stomach, insulin metabolism, male fertility, immune system, bone marrow, and fat.

Regarding osteoporosis studies, P. Jackuliak and J. Payer analyzed the osteoporosis risk in diabetic patients. Moreover the authors made clinical considerations and discussed the use of bone mineral density and the trabecular bone score to study different bone properties, quantity and quality, respectively. M. Bolanowski et al. investigated how many hormonal disorders such as Cushing's syndrome, hyperprolactinemia, acromegaly, hypogonadism, and hypopituitarism influence bone metabolism and can result in secondary osteoporosis.

This special issue should be of interest for basic and clinical researchers since it covers a wide range of topics regarding bone research. Original studies and reviews have been published with the aim to report and summarize the latest findings regarding the relevance of the skeleton in the whole body physiology.

